# Nut consumption in relation to long‐term risk of all‐cause dementia: findings from three prospective cohort studies

**DOI:** 10.1002/alz70860_101501

**Published:** 2025-12-23

**Authors:** Mengjia Zhao, Minqing Yan, Hui Chen, Leqi Fei, Minyu Wu, Jie Shen, Liyan Huang, Changzheng Yuan

**Affiliations:** ^1^ Zhejiang University School of Medicine, Hangzhou, Zhejiang, China; ^2^ Zhejiang University, Hangzhou, Zhejiang, China; ^3^ Harvard T. H. Chan School of Public Health, Boston, MA, USA

## Abstract

**Background:**

Dementia poses a significant health threat to older adults, making preventive strategies critical. Recent research suggests that nut consumption may have beneficial impacts on brain health, although existing evidence is still limited.

**Method:**

We analyzed data from older adults aged 45 and above from the Health and Retirement Study (HRS) in 2013, the Framingham Offspring Study (FOS) in 1998‐2001, and the Whitehall II study (WHII) in 2002‐2004, all of whom were free of dementia at the study baseline. Consumption of nuts (including tree nuts and peanuts) was assessed using validated food frequency questionnaires. Incident all‐cause dementia was tracked through a validated algorithm in HRS (up to 2020), panel reviews in FOS (up to 2016), and healthcare record linkages in WHII (up to 2018). The association between nut consumption and incident dementia was estimated using multivariable Cox proportional hazard models.

**Result:**

This study included 6,116 participants from HRS (mean age, 65.8 [SD, 10.0] years; 3,625 females [59.3%]), 3,061 participants from FOS (mean age, 57.5 [SD, 8.1] years; 1,617 females [52.8%]), and 8,234 participants from WII (mean age, 61.2 [SD, 6.1] years; 2,556 females [31.0%]). Over 218,019 person‐years, a total of 1,046 participants (532 in HRS, 308 in FOS, and 206 in WHII) developed incident dementia. After multivariable adjustment, higher nut consumption was associated with a lower risk of dementia, with pooled hazard ratios (HRs) of 0.79 (95% confidence interval [CI]: 0.70‐0.89; *I* ^2^ = 0%) for 0.1‐5.0 g/day vs. 0 g/day, and 0.76 (0.60‐0.97; *I* ^2^ = 54%) for >5.0 g/day vs. 0 g/day. The observed association was similar across major subgroups.

**Conclusion:**

Our findings indicate that higher nut consumption is associated with a lower risk of incident dementia in a dose‐response manner among middle‐aged and older adults. This suggests that consumption of nuts may serve as an effective preventive strategy for promoting brain health.

